# Stress Granule Dysregulation in Amyotrophic Lateral Sclerosis

**DOI:** 10.3389/fncel.2020.598517

**Published:** 2020-11-17

**Authors:** Jessica Dudman, Xin Qi

**Affiliations:** Department of Physiology and Biophysics, School of Medicine, Case Western Reserve University, Cleveland, OH, United States

**Keywords:** amyotrophic lateral sclerosis, stress granules, protein aggregation, neurodegeneration, therapeutics

## Abstract

Amyotrophic lateral sclerosis (ALS) is a progressive neurodegenerative disease with no current cure. ALS causes degeneration of both upper and lower motor neurons leading to atrophy of the innervating muscles and progressive paralysis. The exact mechanism of the pathology of ALS is unknown. However, 147 genes have been identified that are causative, associated with, or modify disease progression. While the causative mechanism is unknown, a number of pathological processes have been associated with ALS. These include mitochondrial dysfunction, protein accumulation, and defects in RNA metabolism. RNA metabolism is a complicated process that is regulated by many different RNA-binding proteins (RBPs). A small defect in RNA metabolism can produce results as dramatic as determining cell survival. Stress granules (SGs) control RNA translation during stressed conditions. This is a protective reaction, but in conditions of chronic stress can become pathogenic. SGs are even hypothesized to act as a seeding mechanism for the pathological aggregation of proteins seen in many neurodegenerative diseases, including TAR DNA-binding protein 43 (TDP-43) in ALS. In this review, we will be summarizing the current findings of SG pathology in ALS. We also focus on the role of SG dysregulation in protein aggregate formation and mitochondrial dysfunction. In addition, we outline therapeutic strategies that target SG components in ALS.

## Introduction

Amyotrophic lateral sclerosis (ALS) is a devastating, progressive neurodegenerative disease that is universally fatal. ALS is a very heterogeneous disease in both onset and disease progression. Typically ALS starts with focal weakness, pain, or twitching and then spreads to include most muscle groups with increasing paralysis. Eventually the paralysis reaches the diaphragm and death due to respiratory failure typically occurs 3–5 years after diagnosis (Brown and Al-Chalabi, 2017). Most cases begin with asymmetrical limb onset causing leg or arm weakness, pain, or twitching. However, approximately 1/3 of cases begin with bulbar onset which causes problems swallowing, speaking, or chewing (Wijesekera and Nigel Leigh, 2009). There is no current cure, although two disease-modifying therapies have been approved by the FDA (NINDS, 2013). However, these approved strategies only extend life by a few months or slow the progression of functional loss without extending life expectancy.

Amyotrophic lateral sclerosis involves both upper, also called corticospinal, motor neurons and lower motor neurons, which are the neurons that directly innervate the muscles (Brown and Al-Chalabi, 2017). The degree of involvement of each neuron population varies from case to case. Upper motor neuron degeneration causes muscle stiffness and spasticity while lower motor neuron degeneration initially causes twitching while the neurons are dying and then muscle atrophy as the muscle is de-innervated (Brown and Al-Chalabi, 2017). 147 different gene mutations have been found to contribute to ALS pathogenesis (Dervishi et al., 2018). These genes are found in a number of different pathways that potentially contribute to disease progression. These pathways include: axonal transport, the unfolded protein response, endoplasmic reticulum stress (Dervishi et al., 2018), autophagy and mitophagy (Evans and Holzbaur, 2019), the integrated stress response (ISR), nucleo-cytoplasmic trafficking, alternative splicing (Simona et al., 2020), and RNA metabolism (Fan and Leung, 2016).

Stress granules (SGs) are a membraneless organelle in cells that participate in RNA metabolism during times of cellular stress. They are composed primarily of RNA and RNA-binding proteins (RBPs). SGs are an important part of RNA metabolism during times of cellular stress (Wolozin and Ivanov, 2019). Defects in both SG assembly and disassembly have been linked to neurodegenerative disorders (Fan and Leung, 2016; Samir et al., 2019; Wolozin and Ivanov, 2019). SGs are transient structures in a cell that form in response to cell stress. The formation of SGs prevents the formation of NLR Family Pyrin Domain Containing 3 (NLRP3) inflammasomes and protects the cell from undergoing pyroptosis (Samir et al., 2019). The formation of SGs impacts the numbers of other membraneless organelles such as Cajal bodies and nuclear gems (Simona et al., 2020). SGs also inhibit translation during stressed conditions by sequestering RNA. The consequences of chronic cellular stress have not been fully defined, but have been strongly associated with neurodegeneration and other pathologies (Simona et al., 2020). In situations of chronic cellular stress, including aging, SGs can become persistent structures. The chronic presence of SGs can act as a seeding mechanism and lead to the accumulation of RBPs (Wolozin and Ivanov, 2019). In this review, we will focus on SGs, and summarize recent findings of SG dysregulation and its connection with protein aggregations and mitochondrial damage in ALS.

## ALS Genetics

**FIGURE 1:**
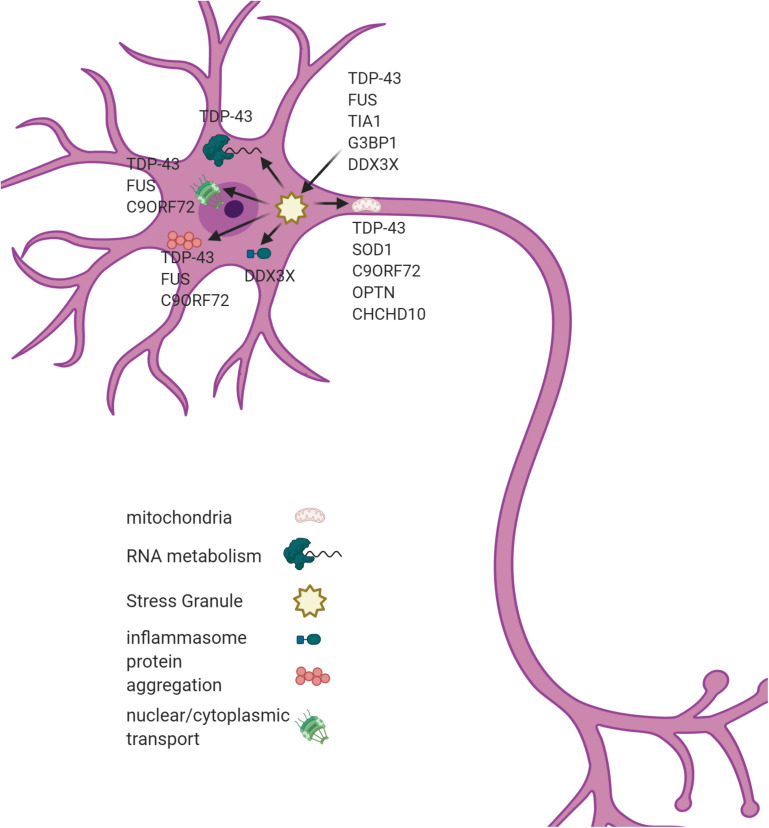
A SGs dysregulation associates with multiple cellular phenotypes in ALS. SGs are hypothesized to act as a seeding mechanism for the pathological aggregations of TDP43 and FUS. SGs also contribute to mitochondrial dysfunction, inflammasome activation, and nucleo-cytoplasmic transport in ALS. Genes associated with each pathological process are listed in the figure. Figure created with BioRender.com.

Though the pathogenesis of ALS remains elusive, analysis of the genetic factors that contribute to ALS has identified some major areas of dysfunction. These include RNA metabolism, protein homeostasis, endoplasmic reticulum stress, and ribonucleoprotein body dynamics (Taylor et al., 2016). A few of the well-known genetic causes of ALS and the effects they have are described here. Figure 1 diagrams various pathways effected in ALS and genes that contribute to their pathology.

The first genetic cause of ALS, mutations in Cu/Zn Superoxide Dismutase (SOD1), was identified in 1993 (NINDS, 2013). SOD1 is a highly expressed enzyme found primarily in the cytosol of cells. The function of SOD1 is to catalyze the conversion of super oxide into hydrogen peroxide and oxygen (Song et al., 2013). SOD1 mutations cause an autosomal dominant form of ALS that has a toxic gain-of-function rather than simply a loss of superoxide scavenging capabilities (Song et al., 2013). The mechanism by which this mutation causes ALS is unclear, although it has been shown to cause mitochondrial dysfunction (Song et al., 2013). The SOD1G93A mutation triggers a decrease in mitochondria length, round fragmented mitochondria, and impaired axonal transport of mitochondria. Mutant SOD1 has been shown to preferentially bind to mitochondria and impair respiration, decrease calcium buffering, block protein import, and induce apoptosis though Bcl-2 (B-cell lymphoma 2) inhibition (Song et al., 2013). In addition to directly causing neurodegeneration, SOD1 mutants elicit alterations in glial functioning (Bravo-Hernandez et al., 2020). Joshi et al. showed that neurotoxic proteins expressed in microglia alone are sufficient to trigger neurodegeneration *in vitro*. When SOD1G93A was expressed in cultured microglia, it caused the release of dysfunctional mitochondria into the extracellular space. When conditioned media from this mutant microglia culture was transferred onto naïve astrocytes, it triggered the astrocytes to enter the A1 proinflammatory state and release fragmented mitochondria of their own as well as inflammatory cytokines (Joshi et al., 2019). Other research has shown that astrocytes in the A1 state are proinflammatory while astrocytes in the A2 state are neuroprotective (Liddelow et al., 2017). Another group showed that mutant SOD1 expression in oligodendrocytes was sufficient to cause hyperexcitability and death in wild type motor neurons (Ferraiuolo et al., 2016).

The C9ORF72 repeat expansion is the most prevalent genetic cause of ALS (Cheng et al., 2019). The C9ORF72 repeat expansion’s toxicity increases with age, repeat length, and expression level (Jiang et al., 2016). There is conflicting evidence as to whether this is due to toxic gain of function or loss of normal C9ORF72 function. Jiang et al. (2016) concluded that this toxicity is due to a gain of function rather than a loss of normal function as C9ORF72 knockout mice developed splenomegaly and enlarged lymph nodes rather than motor deficits. However, Shi et al. (2018) found that C9ORF72 mutations show both gain and loss of function properties as haploinsufficiency leads to defects in vesicle trafficking that lead to an accumulation of glutamate receptors which results in excitotoxicity and neurodegeneration. The C9ORF72 repeat expansion produces dipeptide repeat (DPR) proteins through non-conventional translation mechanisms (Cheng et al., 2019). These DPR proteins have been shown to exhibit toxic effects by interfering with nucleo-cytoplasmic transport and disassembling Cajal bodies and nuclear gems (Simona et al., 2020).

Although mutations in TAR DNA-binding protein 43 (TDP)-43 are less common that SOD1 or C9ORF72, TDP-43 mislocalization to the cytoplasm is a prominent feature in ALS that is seen in both familial and sporadic ALS (Brown and Al-Chalabi, 2017). 5–10% of familial cases of ALS have mutations in TDP-43 and despite lacking known mutations 97% of sporadic cases have TDP-43 inclusion bodies in their brain and spinal cord (Prasad et al., 2019). The exact molecular mechanism by which cytoplasmic TDP-43 results in neurodegeneration is unclear, but it has been shown to accumulate in the mitochondria (Wang et al., 2016), impair overall nucleo-cytoplasmic trafficking (Simona et al., 2020), and associate with SGs (Khalfallah et al., 2018). Once localized to the mitochondria TDP-43 binds mitochondrial transcribed mRNA and impairs the expression of complex I proteins causing mitochondrial dysfunction (Wang et al., 2016). Simply overexpressing TDP-43 in cell culture will result in the formation of and colocalization with SGs without any other stressor (Fan and Leung, 2016).

## Stress Granules

The response to stress by the RNA metabolism machinery has been strongly indicated in the pathology of neurodegeneration, particularly in ALS. SGs are a type of ribonucleoprotein body that assembles through liquid–liquid phase separation (Taylor et al., 2016). TDP-43, the protein that forms inclusions in ALS, has been shown to undergo liquid-liquid phase separation *in vivo* (Prasad et al., 2019). SGs are meant to be transient structures, but chronic stresses can cause persistent SGs. These long-term SGs appear to function as a nidus for the aggregation of pathological proteins. Aging, among other things, places cells under chronic stress (Khalfallah et al., 2018). When this occurs in a patient with non-ideal RNA metabolism machinery, this could cascade into a neurodegenerative disease (Wijesekera and Nigel Leigh, 2009).

RNA are rarely found alone in a cell; instead, they form ribonucleoprotein complexes with other RNA and RBPs (Fan and Leung, 2016). These complexes can in turn aggregate and form RNA granules through liquid–liquid phase separation. There are many types of RNA granules including: nucleoli, Cajal bodies, nuclear speckles and paraspeckles, P-bodies, and SGs (Fan and Leung, 2016). Activation of the ISR causes the reorganization of many of the membraneless organelles found within the cell (Simona et al., 2020). It increases the number of SGs and induces a variety of proteins (mediators of N/C transport, importin-B1) to relocate to and accumulate in SGs (Simona et al., 2020). Interestingly, the overexpression of importin-B1 can significantly reduce the number of SGs created in response to stress (Simona et al., 2020).

RNA granules are unique structures due to their ability to segregate RNA and protein into a membrane-less organelle. SGs are also unique among RNA granules due to their transient nature. Typically SGs form in response to some sort of cellular stress, oxidative stress, for example, and disassemble once conditions return to normal. Mammalian SGs are spheroid or ellipsoid and can range from 0.4 to 5 μm in diameter (Fan and Leung, 2016). SGs can be visualized under a microscope and morphologically similar structures have been observed in the neurons of patients with ALS and other neurodegenerative disorders (Fan and Leung, 2016). A defining feature of SGs is the presence of stalled 48S pre-initiation complexes which form the core SG components. A second class of proteins are added after the initiation event. Interestingly, when these secondary proteins, T-Cell-Restricted Intracellular Antigen-1 (TIA-1), for example, are overexpressed, they will spontaneously induce SG formation even in the absence of a stressor. There is another tertiary group of proteins that may not have a role in RNA metabolism, but it has been suggested that they allow SGs to function in cell signaling. The exact composition of SGs is variable and dependent on the triggering stressor (Fan and Leung, 2016).

Khalfallah et al. investigated the differences in SG assembly and disassembly in different cell populations relevant to ALS. This study was undertaken due to the fact that the TDP-43, ubiquitin positive inclusions frequently seen in patients are found in some glia as well as neurons. SG morphology and composition differ depending on both the cell type they are induced in as well as the stressor used to induce them. Cortical neurons took about twice as long to develop SGs as astrocytes and the disassembly of those SGs also took about twice as long in neurons as in astrocytes (Khalfallah et al., 2018).

It is hypothesized that the chronic stress of aging can lead to the formation of chronic SGs that then act as a nidus for the aggregation of disease associated proteins (Wolozin and Ivanov, 2019). This hypothesis is founded on the fact that the pathological TDP-43 inclusions found in ALS patient samples also colocalize with markers for SGs (Wolozin and Ivanov, 2019). SGs have been well characterized under conditions of acute stress, heat shock, for example, but less work has been done to analyze SG assembly during chronic stress (Reineke and Neilson, 2019; Wolozin and Ivanov, 2019; Simona et al., 2020). The typical lab experiment to analyze SGs involves 30–60 min of severe stress. In contrast, ALS typically lasts 3–6 years. A persistent mild stressor could allow cells to survive with SGs, but the additional time without disassembly could allow those SGs to mature into pathologically stable inclusions. The low complexity regions in many RBPs are important for the liquid–liquid phase separation of SGs, but they can also form beta-sheets that accumulate into amyloids (Wolozin and Ivanov, 2019). Continued stress has also been shown in live cells to cause TDP-43 associated SGs to become less dynamic and form non-fluid gels (Wolozin and Ivanov, 2019). Acute and chronic SGs have slightly different protein compositions and lead to very different cell fate decisions. Acute SGs contain signaling components and 40S ribosomes. They are very dynamic and have a pro-survival function. Chronic SGs, in contrast, are static structures that include different proteins and have a pro-death function (Reineke and Neilson, 2019). For example, chronic SGs contain the protein Fused in Sarcoma (FUS), which is another protein that has been associated with ALS. Chronic SGs do not also contain 40S ribosomes which could indicate that the cell has lost the ability to translate the RNA within the SG (Reineke and Neilson, 2019).

Defects in SG assembly and disassembly have already been linked to a number of neurodegenerative disorders including ALS. TDP-43 and FUS are RBPs that colocalize with SG markers in the cytoplasmic inclusions commonly found in ALS patient samples. Many of the mutations known to contribute to ALS disrupt TDP-43 and FUS shuttling between the nucleus and cytoplasm, or increase their aggregation ability. Additionally, TDP-43 and FUS mislocalization effects the cells entire nucleo-cytoplasmic transport capability (Simona et al., 2020). Either of these disruptions can lead to toxic inclusions (Fan and Leung, 2016). In fact, simply overexpressing TDP-43 or expressing the truncated form commonly found in ALS patients will trigger colocalization with SG markers even in the absence of stress (Fan and Leung, 2016). TDP-43 levels contribute to SG assembly by regulating the levels of nucleating RBPs TIA-1 and GTPase Activating Protein (SH3 Domain) Binding Protein 1 (G3BP1) (Fan and Leung, 2016).

The formation and presence of SGs by any method is sufficient to modulate nucleo-cytoplasmic transport (Simona et al., 2020). The mere presence of SGs also directly inhibits the formation of the NLRP3 inflammasome through competitive sequestering of DEAD-Box Helicase 3 X-Linked (DDX3X). In this way, free DDX3X acts as a “live-or-die signal,” sequestered in SGs it supports cell survival and when bound to the NLRP3 inflammasome complex it leads to pyroptosis (Samir et al., 2019). However, persistent cytoplasmic inclusions that contain SG components have been linked to neurodegeneration (Fan and Leung, 2016).

If the presence of SGs is sufficient to cause pathology, then a malfunction in the disassembly of SGs would be an additional source of pathology. Additional ALS associated mutations have been found in Valosin-containing protein (VCP) which suggests a protective role for heat shock proteins in SG disassembly (Fan and Leung, 2016). RBPs typically contain prion like or low complexity domains that are important for SG liquid–liquid phase separation, but need to be kept in check by chaperones to avoid pathological aggregation (Fan and Leung, 2016). This balance between disorder and regulation is a delicate one that leaves SG assembly and disassembly processes vulnerable to disruption (Fan and Leung, 2016).

## Protein Aggregation and Stress Granules

Most neurodegenerative diseases are characterized by the aggregation of some particular protein, in ALS that protein is TDP-43. There is evidence to suggest that there is intercellular transmission of these pathologically misfolded proteins which is known as “pathological seeding.” Progress is being made on analyzing the methods of cell-to-cell transmission of these aggregates (Peng et al., 2020). Additional support for “potential prion-like propagation” of TDP-43 has also been shown by Prasad et al. (2019). Cytoplasmic mislocalization and aberrant post-translational modifications of TDP-43 occur in ALS. The hyperphosphorylated and ubiqutinated TDP-43 form deposits known as inclusion bodies in the majority of ALS patients. *In vitro* experiments show TDP-43 forming amyloid like aggregates and *in vivo* experiments show pathological oligomerization (Prasad et al., 2019).

RNA helicases, like DDX3X, are vital for nearly every aspect of RNA metabolism. They are ubiquitous and highly conserved enzymes that bind and remodel RNA in an ATP-dependent manner (Jankowsky, 2011). DDX3X is an RNA helicase that has been shown to bind to the repeat expanded region in C9ORF72. By binding the repeat regions, DDX3X prevents the non-AUG translation of these expanded repeats. A non-traditional method of translation that has been identified is called repeat-associated non-AUG (RAN) translation (Gao et al., 2017). RAN translation occurs by an unknown mechanism, but DDX3X binding would mask the repeat region of C9ORF72. This is hypothesized to prevent the formation of DPR proteins and their aggregation (Cheng et al., 2019).

## Mitochondrial Dysfunction and Stress Granules

Mitochondrial dysfunction and fragmentation is widely observed in many different neurodegenerative disease models (Guo et al., 2013; Su and Qi, 2013; Joshi et al., 2019). Mutations in genes believed to be in the autophagy/mitophagy pathway are known to cause neurodegenerative diseases including ALS (Evans and Holzbaur, 2019). One example is optineurin (OPTN) which is implicated in ALS and Parkinson’s disease (Monahan et al., 2016). OPTN binds the autophagosome receptor LC3 and facilitates mitophagy. The ALS linked mutation OPTNE478G is in the ubiquitin-binding domain and mutations in this domain cause reduced autophagic ability (Monahan et al., 2016). This is not only a problem in neurons, ALS proteins expressed solely in microglia or astrocytes increase the ratio of damaged to functional mitochondria that are released into the extracellular matrix which in turn is sufficient to cause neurodegeneration in a cell culture model (Joshi et al., 2019). Additionally, CHCHD10 is a mitochondrial protein that when mutated disrupts cristae and mitochondrial structure and causes ALS (Smith et al., 2019).

In addition to forming toxic DPR protein aggregates, the poly-GR produced by the C9ORF72 repeat expansion can bind complex V of the mitochondrial respiratory chain and increase its degradation leading to impaired mitochondrial dynamics (Choi et al., 2019). Other ALS associated mutant proteins are also known to accumulate in the mitochondria, and cause damage to mitochondrial function on many different levels (Smith et al., 2019). SOD1 aggregates in mitochondria, decreases ATP generation, increases ROS damage, causes an imbalance in Ca homeostasis, disrupts mitochondrial architecture, ER-mitochondrial contacts, dynamics, and transport, and induces apoptosis. TDP-43 also aggregates in mitochondria, disrupts mtDNA transcription, decreases ATP production, disrupt Ca homeostasis, ER-mitochondrial contacts, architecture, network dynamics, axonal transport, and reduces mitophagy (Smith et al., 2019).

Mitochondrial stress is also sufficient to cause SG formation (Monahan et al., 2016). Overexpression of wild type or ALS mutant TDP-43 in motor neuron-like NSC-34 cells causes increased ROS production and oxidative damage, whereas under normal cell conditions, TDP-43 plays a protective role in oxidative stress which will trigger its recruitment to SGs (Smith et al., 2019).

## Potential Therapeutic Approaches

Despite all of the research that has been done, there is no cure for ALS. The FDA has approved the drugs riluzole (Rilutek) and edaravone (Radicava) to treat ALS. Riluzole has been shown to extend life by a few months and edaravone slows the decline in daily functioning (NINDS, 2013). Analysis of converging mutation pathways could open up new avenues of therapeutics (Dervishi et al., 2018). Some recent studies propose new techniques and targets that have the potential for new therapeutic targets, although more research is necessary.

Gene silencing is a potential therapeutic using shRNA expressed by an adeno-associated virus (AAV) injected into the subpial spinal cord. This has been shown to provide robust dissemination of the AAV expressing the shRNA throughout the CNS. Treatment using an AAV expressing a shRNA targeted to the mutant SOD1 in a SOD1 mutant ALS mouse model has been show to prevent or stop the progression of ALS depending on the timing of the injection (Bravo-Hernandez et al., 2020). This treatment has not yet been tested for efficacy in non-human primates, but the subpial AAV injection technique has been tested for dissemination using a GFP expressing AAV which was able to be found throughout the CNS in non-human primates and pigs (Bravo-Hernandez et al., 2020). This technique shows promise for the familial forms of ALS, but its applicability to the more common sporadic form of ALS is unclear.

Reducing the production of DPR proteins is another potential therapeutic target. Elevating DDX3X expression has been shown to be sufficient to rescue neuron survival in patient iPSC culture (Cheng et al., 2019). An alternative method to reducing DPR uses antisense oligonucleotides (ASO) against the expanded repeat region in C9ORF72. This can also induce a decrease in repeat RNA while potentially maintaining levels of the wild type RNA. Treatment with these ASOs showed an improved phenotype *in vivo* indicating its possibility as a therapeutic target (Jiang et al., 2016).

Treating the mitochondrial dysfunction is also a potential therapeutic target for ALS (Song et al., 2013). It is possible that a therapeutic that can prevent the release of damaging dysfunctional mitochondria while still allowing the neuroprotective effects of intact mitochondrial release could be sufficient to slow or prevent disease progression (Joshi et al., 2019). An additional mitochondrial pathology seen is defects in mitophagy, which have also been observed in ALS. This opens up an alternative potential avenue of treatment. However, therapeutically manipulating such a widespread and essential cellular process comes with additional risks and complication (Evans and Holzbaur, 2019). Experiments that simply increase autophagy in SOD1 mutant mice actually had a deleterious effect on motor neuron survival and symptom progression. The conclusion reached by these researchers was that while this is a viable pathway for therapeutic targeting, better understanding of the biology of autophagy and mitophagy is needed to more specifically target a therapeutic intervention (Evans and Holzbaur, 2019).

Inflammasome assembly can cause cellular damage and death. Therapeutics targeting the NLRP3 inflammasome have been pursued. There have also been a number of other factors identified that play a role in the activation or deactivation of the NLRP3 inflammasome that could be therapeutically targeted for a more pathology specific treatment. This would include DDX3X and potentially other SG proteins (Liu et al., 2020).

Cytoplasmic aggregation of TDP-43 is a common finding in ALS patients. Although the pathological impact of these aggregations is not entirely known, there are a number of potential mechanisms with supporting evidence. This indicates that therapeutics targeting TDP-43 inclusions for disaggregation could potentially be an effective treatment strategy (Prasad et al., 2019). Since many other neurodegenerative disorders are characterized by abnormal protein aggregation, this type of therapeutic would potentially lead to the development of addition treatments for a wider variety of diseases.

## Discussion

Despite extensive study and a great deal of progress into understanding the pathological hallmarks of ALS, the exact mechanism of neurodegeneration and a cure have remained elusive. A number of recent studies have pointed to dysfunctional RNA metabolism, protein aggregation, and mitochondrial dysfunction as three potential causes, not just for ALS but for many neurodegenerative diseases. It is also likely that these seemingly disparate pathologies are linked to one another. There are still many gaps in our knowledge of neurodegenerative diseases. A greater understanding of why neuronal subtypes are susceptible to specific insults is one current gap in our knowledge. Analyzing the protein products and interactions of known mutations has been used to identify functional pathways that are impacted in ALS (Dervishi et al., 2018). But this still has not fully explained why the motor neurons selectively degenerate in ALS, particularly when it has been shown that neurotoxic proteins expressed solely by glia are sufficient for neurodegeneration (Joshi et al., 2019). There have been no current studies directly investigating the role that SGs play in ALS. However, numerous studies have identified SG components as having an impact on ALS pathology. Even more studies have been performed showing that protein aggregation is a hallmark of not only ALS, but many other neurodegenerative diseases. These pieces of information indicate SG pathology may be a fruitful area for future studies.

## Author Contributions

JD summarized the literature and drafted the manuscript. XQ edited the manuscript. All authors contributed to the article and approved the submitted version.

## Conflict of Interest

The authors declare that the research was conducted in the absence of any commercial or financial relationships that could be construed as a potential conflict of interest.
